# Supplementary material for: Hematopoietic chimerism and donor-specific skin allograft tolerance after non-genotoxic CD117 antibody-drug-conjugate conditioning in MHC-mismatched allotransplantation
Source: Nat Commun. 2019 Feb 6;10:616. doi: 10.1038/s41467-018-08202-w (PMC6365540; doi:10.1038/s41467-018-08202-w)
Supplement: Supplementary file 1 — Supplementary Information [file 41467_2018_8202_MOESM1_ESM.pdf]

Hematopoietic Chimerism and Donor-Specific Skin Allograft Tolerance after Non-Genotoxic CD117 Antibody-Drug-Conjugate Conditioning in MHC-Mismatched Allotransplantation

Li et al

Supplementary information

Supplementary Figure 1

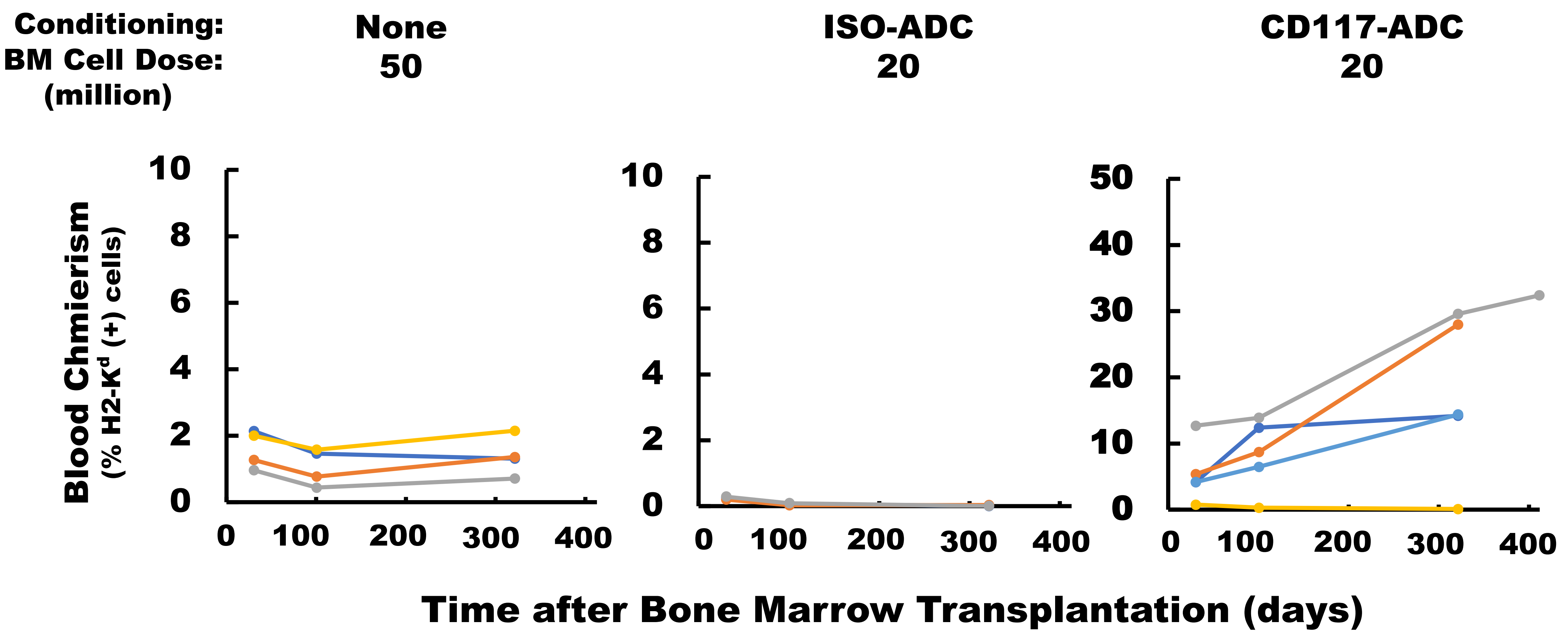

Robust hematopoietic chimerism after CD117-ADC conditioning and transient immunosuppression in fully MHC-mismatched BM allotransplantation: time course. The protocol is given in Figure 1A. The conditioning agent and donor bone marrow cell dose are indicated at the top for each graph. ADC, antibody-drug-conjugate; CD117, anti-CD117 mAb; ISO, isotype control mAb. Each line graph represents an individual mouse from a single experiment confirming results shown in Figure 1B. Results are from one representative experiment different from that represented in Figure 1C.

Supplementary Figure 2

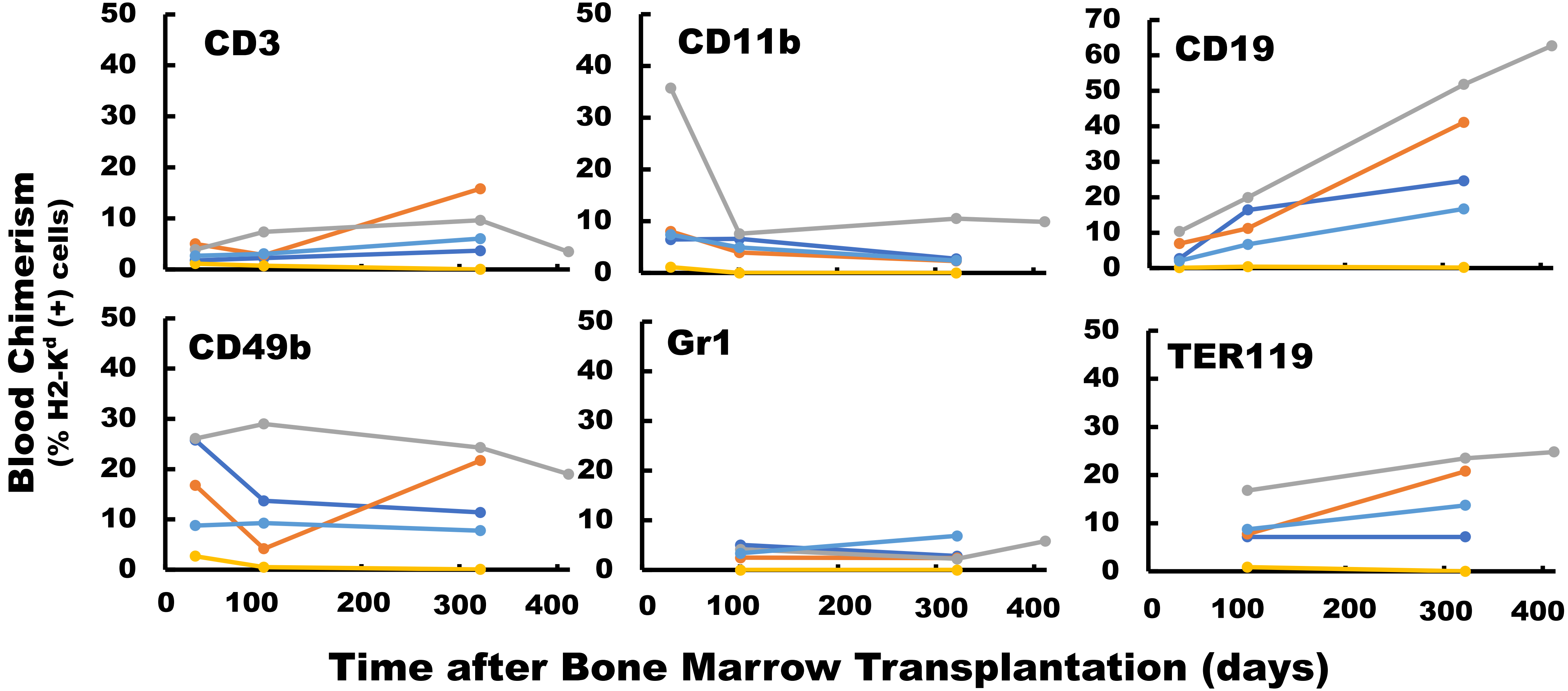

Robust hematopoietic chimerism after CD117-ADC conditioning and transient immunosuppression in fully MHC-mismatched BM allotransplantation: time course of mature blood cell subsets. The protocol is given in Figure 1A. Each line graph is color coded to represent an individual mouse for each of the subsets identified at the top of each panel from a single experiment. No hematopoietic chimerism was observed for ISO-ADC control-conditioned mice (Figure 1B and Supplementary Figure 1).

Supplementary Figure 3

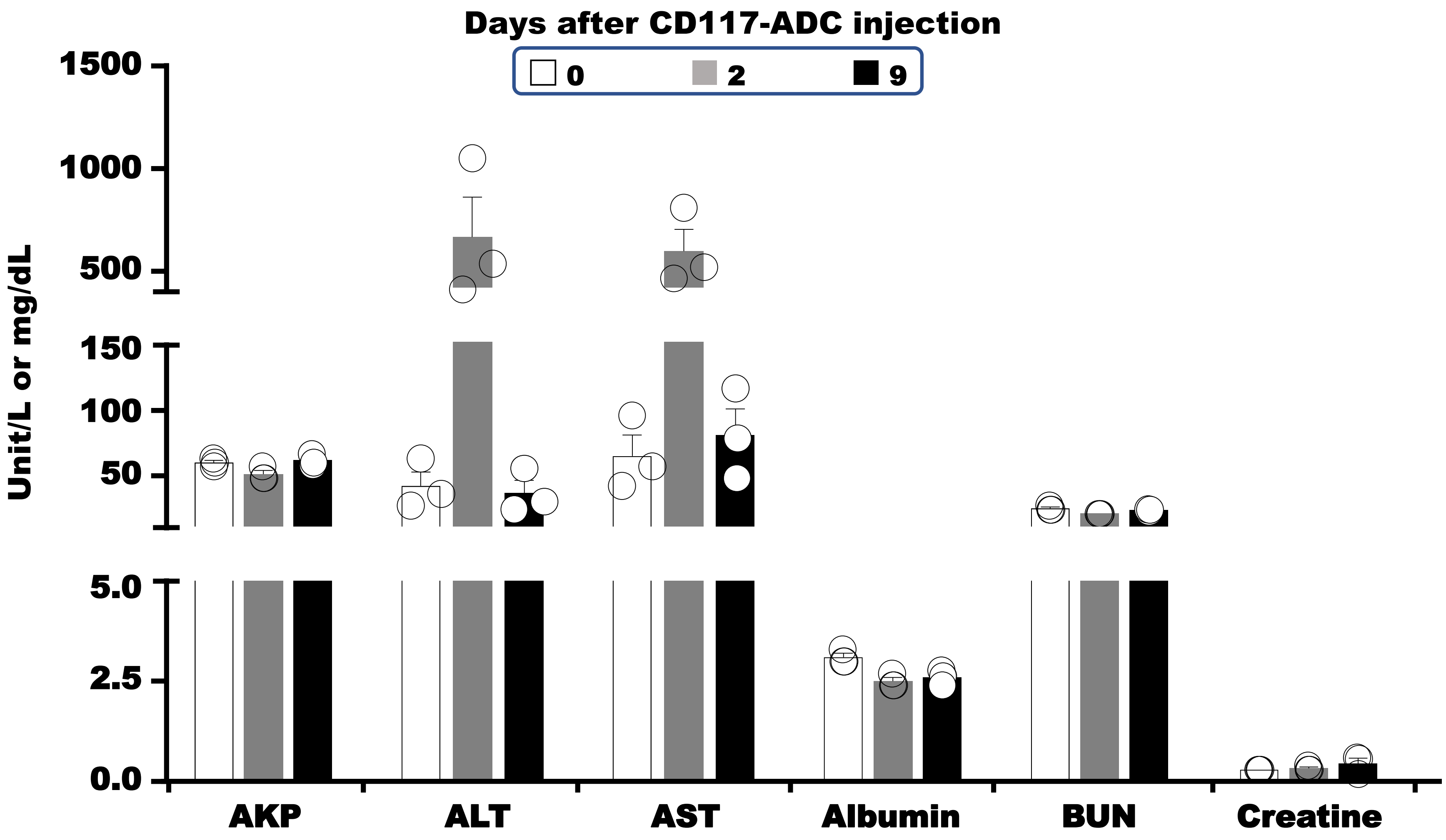

Transient liver toxicity after CD117-ADC conditioning in recipient mice. Liver and kidney function tests were performed on serum obtained on the indicated days after injection of CD117-ADC. The data from a single experiment (n=3) are shown. AKP: alkaline phosphatase, ALT: alanine transaminase, AST: aspartate transaminase, BUN: blood urea nitrogen. AKP, ALT and AST were measured in Unit/L, while Albumin, BUN and Creatine were measured in mg/dL.

Supplementary Figure 4

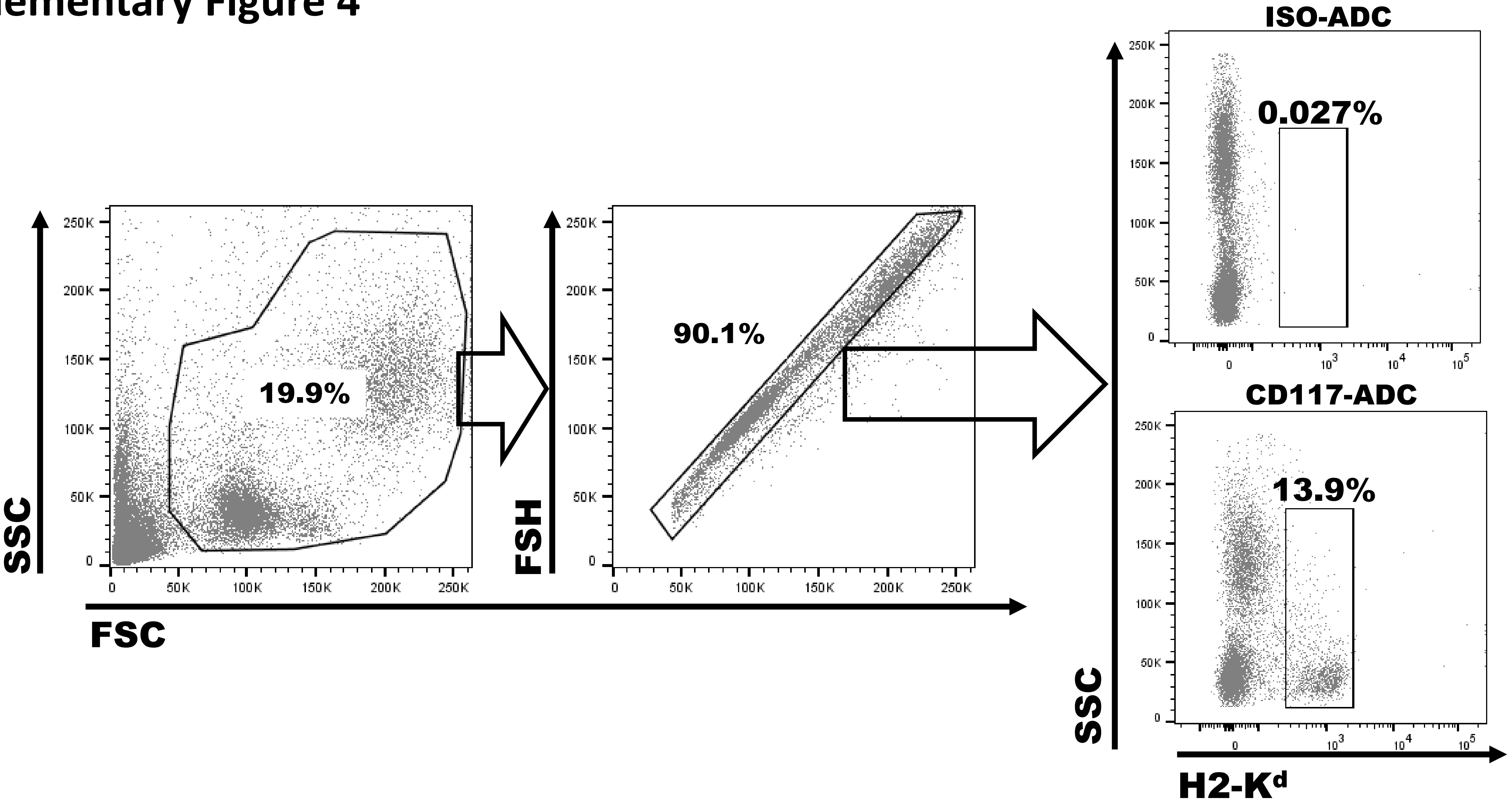

Gating strategy for chimerism analysis in blood. The gating strategy is shown for analyzing the percentage of H2-K^d (+) cells in peripheral blood 100 ~ 600 days after bone marrow transplantation of 20 million bone marrow cells, shown in Figures 1B & 1C, and Supplementary figures 1 & 2. ADC: antibody-drug-conjugate, CD117, anti-CD117 mAb; ISO, isotype control mAb.

Supplementary Figure 5

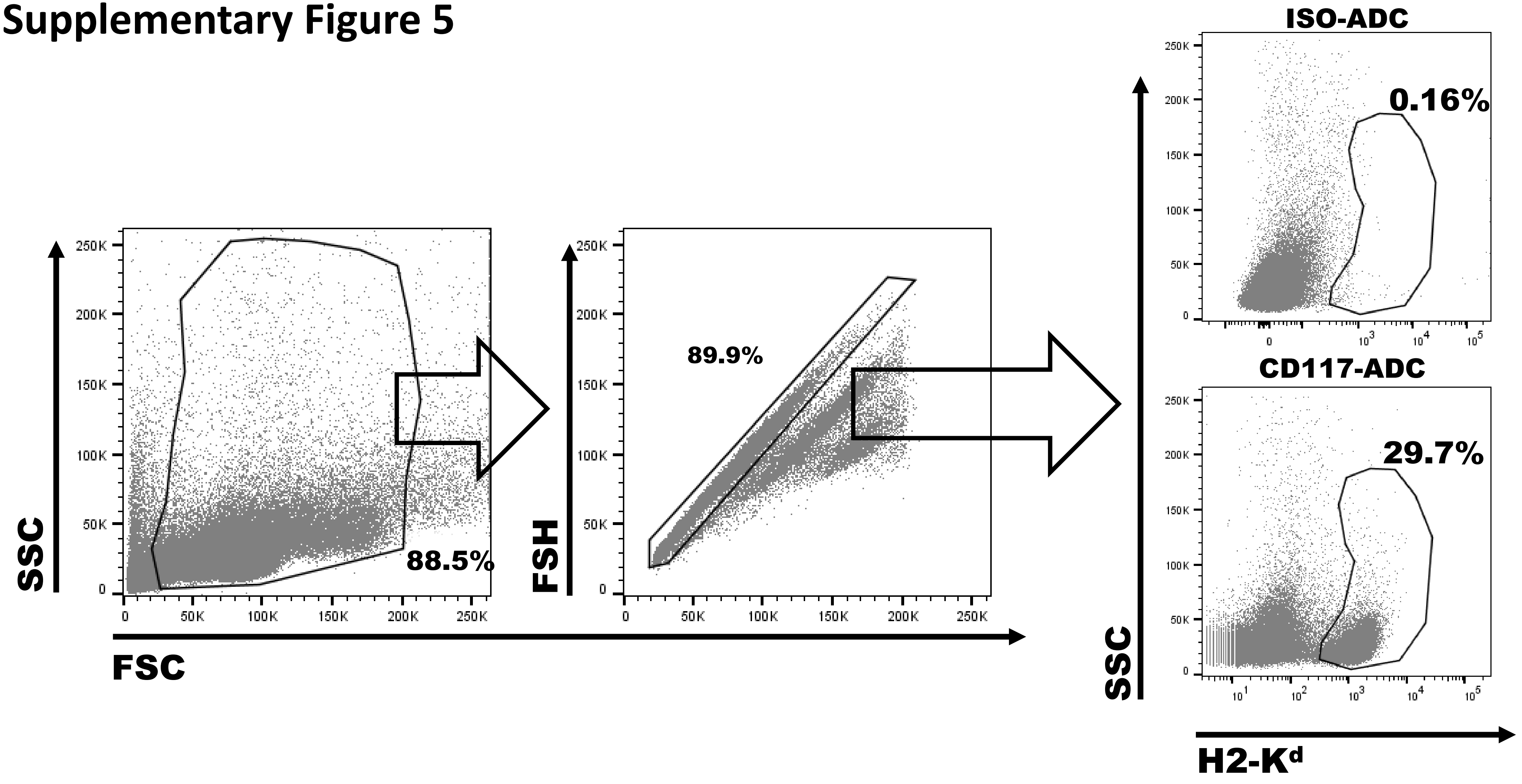

Gating strategy for chimerism analysis in spleen and other immune organs. The gating strategy is shown for analyzing the percentage of H2-K^d (+) cells in immune organs 500 days after bone marrow transplantation of 20 million bone marrow cells, shown in Figure 1D. ADC, antibody-drug-conjugate, CD117, anti-CD117 mAb; ISO, isotype control mAb.

Supplementary Figure 6

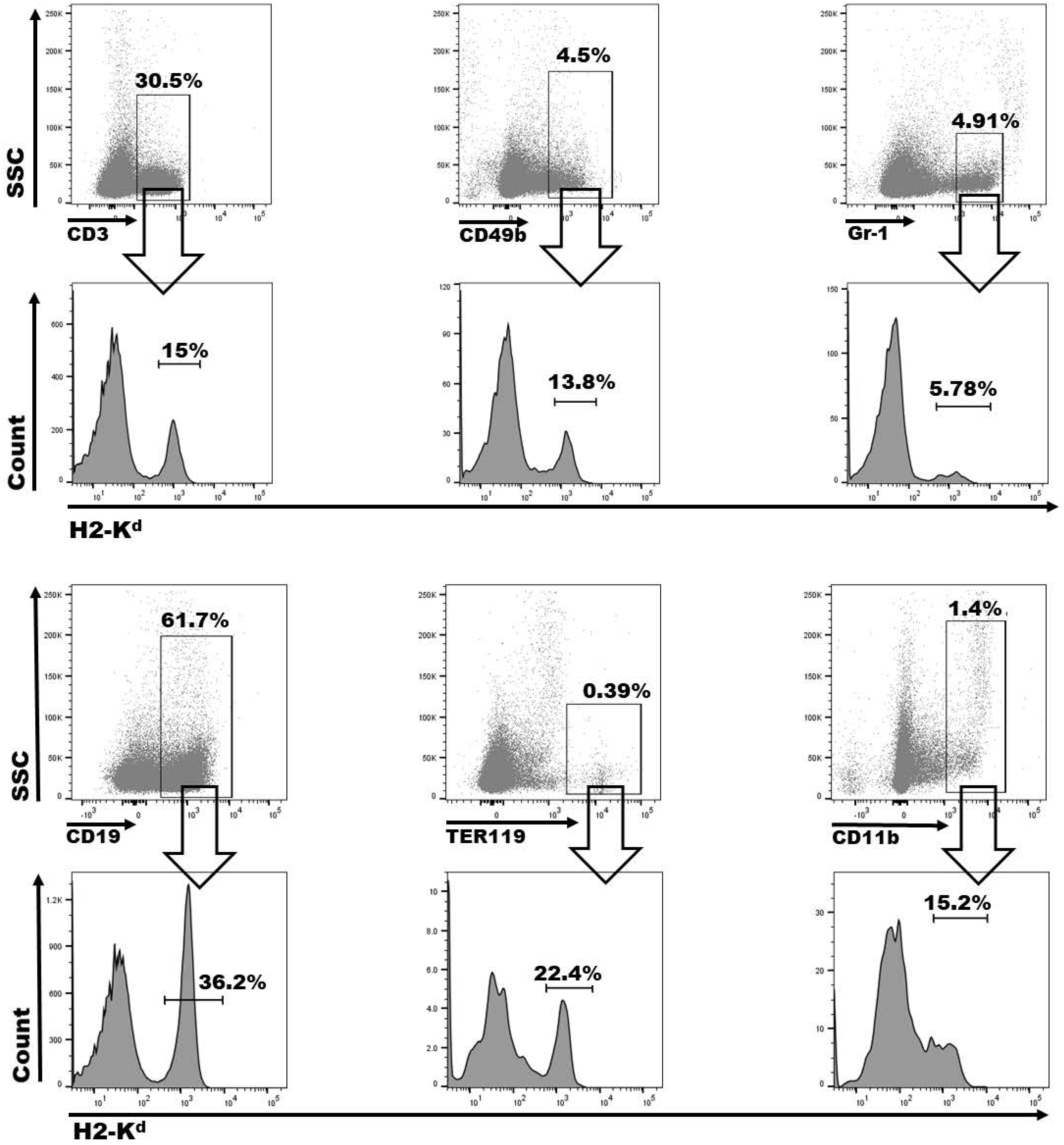

The gating strategy is indicated for analyzing the percentage of H2-K^d (+) cells in the different immune cell subsets shown in Figure 1E.

Supplementary Figure 7

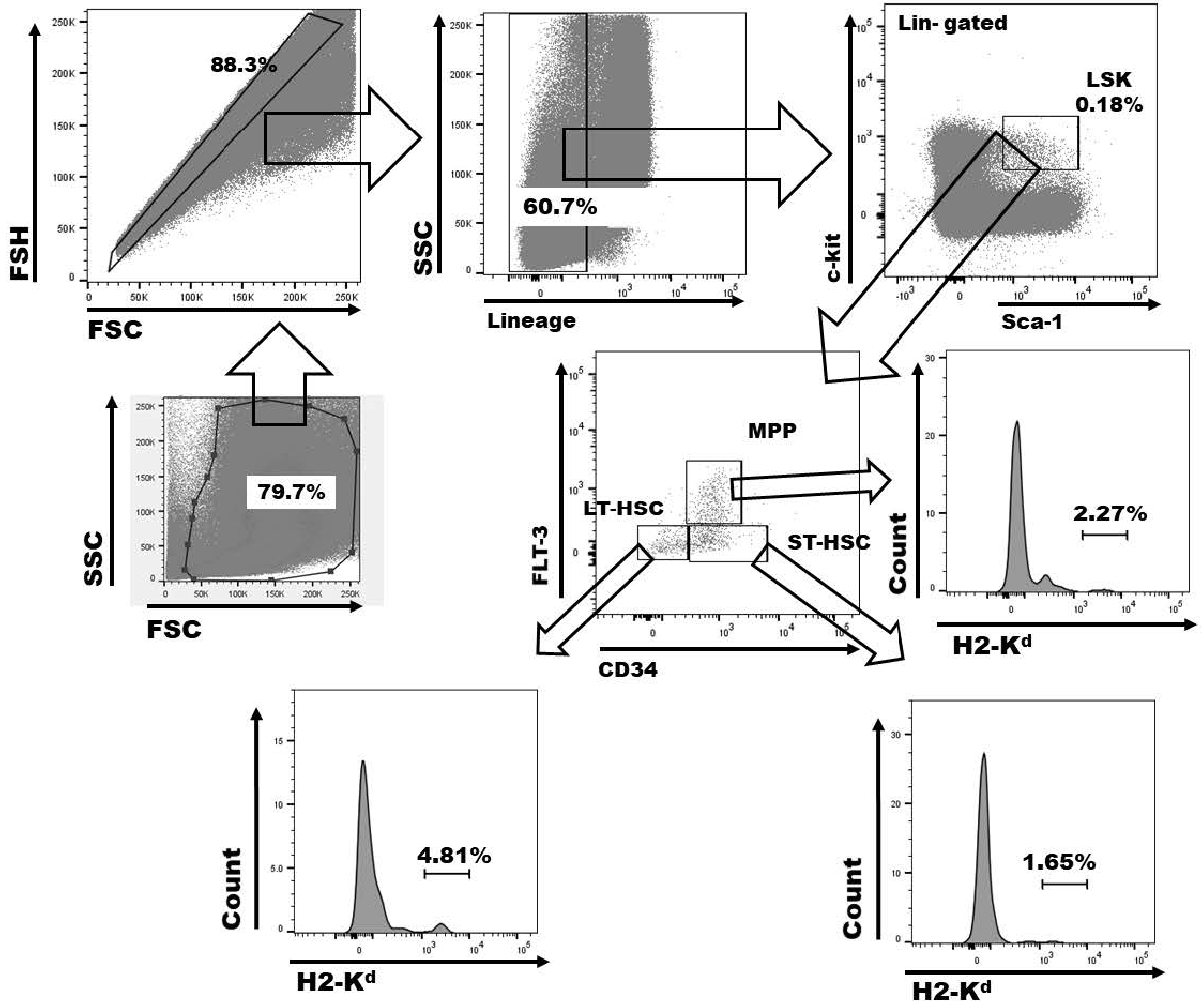

The gating strategy is indicated for analyzing the percentage of H2-K^d (+) cells in the different bone marrow progenitor cell subtypes shown in Figure 1F.

Supplementary Figure 8

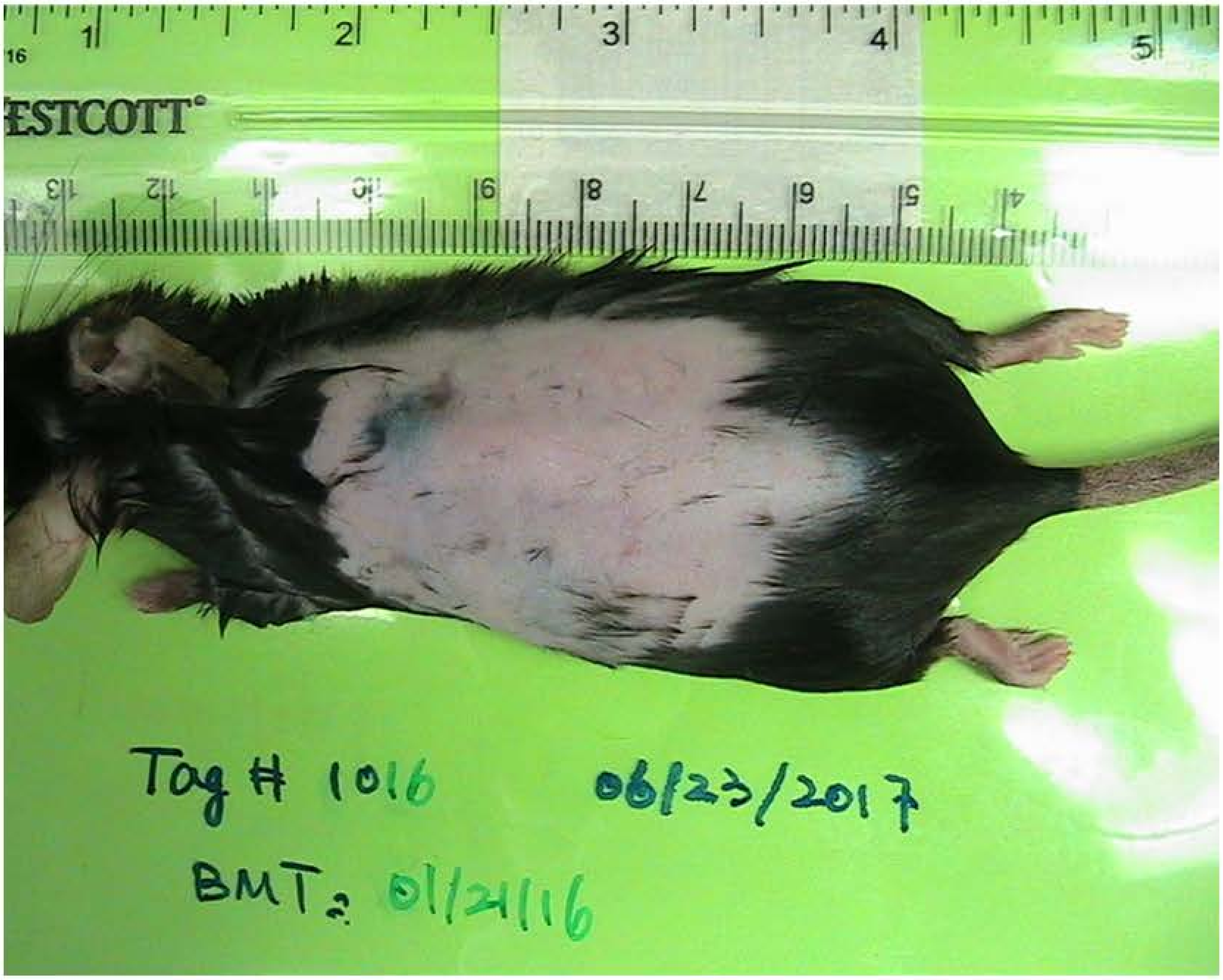

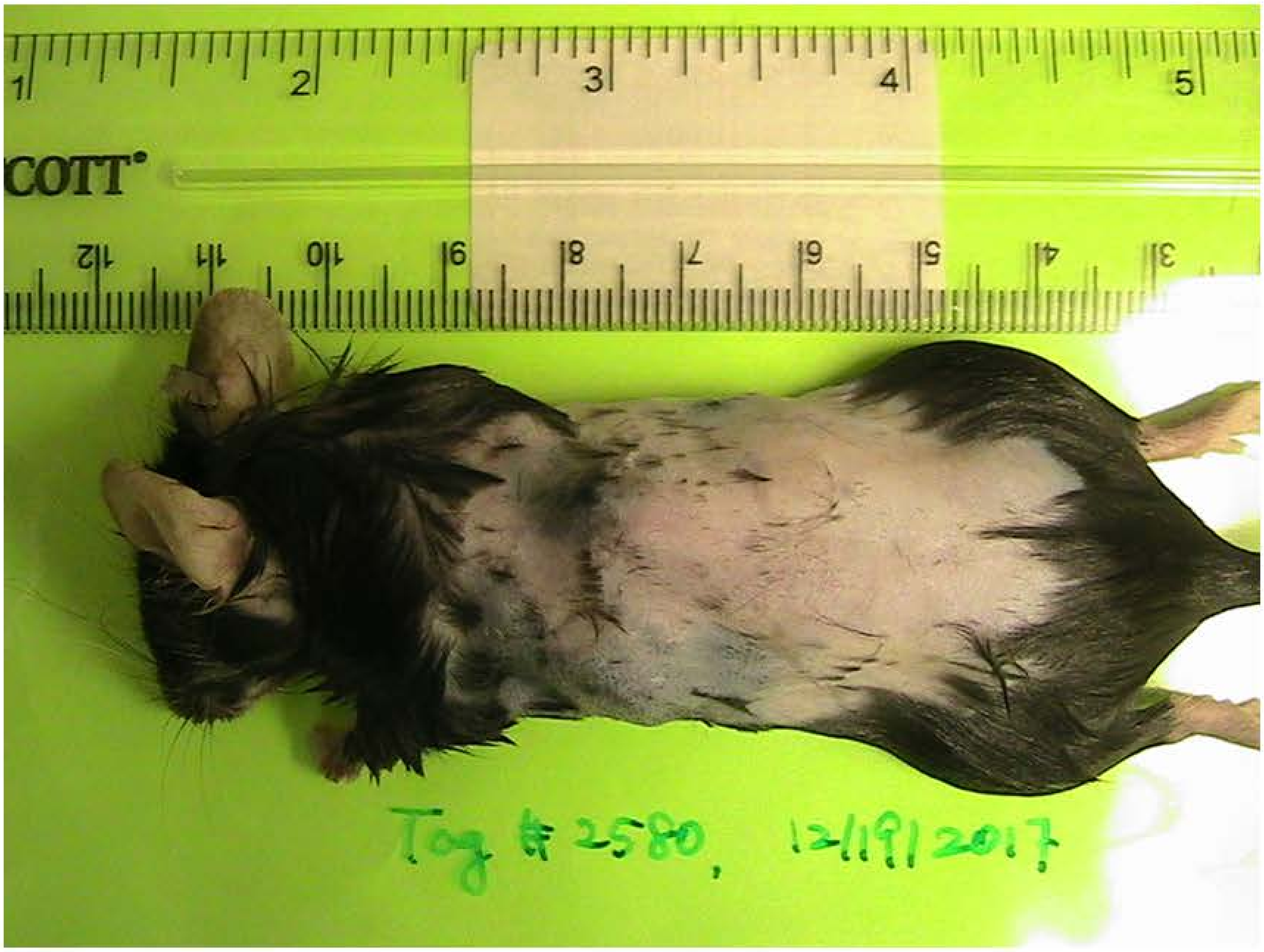

Uncropped images of the pictures shown in Figure 2A.
