# Supplementary material for: Hematopoietic chimerism and donor-specific skin allograft tolerance after non-genotoxic CD117 antibody-drug-conjugate conditioning in MHC-mismatched allotransplantation
Source: Nat Commun. 2019 Feb 6;10:616. doi: 10.1038/s41467-018-08202-w (PMC6365540; doi:10.1038/s41467-018-08202-w)
Supplement: Supplementary file 3 — Reporting Summary [file 41467_2018_8202_MOESM3_ESM.pdf]

nature research

Corresponding author(s): *Double-blind peer review submissions: write DBPR and your manuscript number here instead of author names.*

Reporting Summary

Nature Research wishes to improve the reproducibility of the work that we publish. This form provides structure for consistency and transparency in reporting. For further information on Nature Research policies, see Authors & Referees and the Editorial Policy Checklist.

Statistical parameters

When statistical analyses are reported, confirm that the following items are present in the relevant location (e.g. figure legend, table legend, main text, or Methods section).

n/a	Confirmed	
☐	☒	The exact sample size (n) for each experimental group/condition, given as a discrete number and unit of measurement
☐	☒	An indication of whether measurements were taken from distinct samples or whether the same sample was measured repeatedly
☐	☒	The statistical test(s) used AND whether they are one- or two-sided<br>*Only common tests should be described solely by name; describe more complex techniques in the Methods section.*
☒	☐	A description of all covariates tested
☐	☒	A description of any assumptions or corrections, such as tests of normality and adjustment for multiple comparisons
☐	☒	A full description of the statistics including central tendency (e.g. means) or other basic estimates (e.g. regression coefficient) AND variation (e.g. standard deviation) or associated estimates of uncertainty (e.g. confidence intervals)
☐	☒	For null hypothesis testing, the test statistic (e.g. F, t, r) with confidence intervals, effect sizes, degrees of freedom and P value noted<br>*Give P values as exact values whenever suitable.*
☒	☐	For Bayesian analysis, information on the choice of priors and Markov chain Monte Carlo settings
☒	☐	For hierarchical and complex designs, identification of the appropriate level for tests and full reporting of outcomes
☒	☐	Estimates of effect sizes (e.g. Cohen's d, Pearson's r), indicating how they were calculated
☐	☒	Clearly defined error bars<br>*State explicitly what error bars represent (e.g. SD, SE, CI)*

Our web collection on statistics for biologists may be useful.

Software and code

Policy information about availability of computer code

Data collection	All single cell data were collected using an LSRII flow cytometer (BD Biosciences).
Data analysis	FACS data were analyzed with FlowJoTM 10 software (version 10.2; Treestar, Ashland, OR) using previously published subset definitions (ref. 11). Patterns in the data and differences between groups were analyzed using Prism 7.02 (GraphPad Software).

For manuscripts utilizing custom algorithms or software that are central to the research but not yet described in published literature, software must be made available to editors/reviewers upon request. We strongly encourage code deposition in a community repository (e.g. GitHub). See the Nature Research guidelines for submitting code & software for further information.

Data

Policy information about availability of data

All manuscripts must include a data availability statement. This statement should provide the following information, where applicable:

- Accession codes, unique identifiers, or web links for publicly available datasets
- A list of figures that have associated raw data
- A description of any restrictions on data availability

All data generated and analysed during this study are included in this published article (and its supplementary information files). Two files for source data are

provided. The first file contains data underlying Fig 1B through 1F, Fig 2B and supplementary figures 1 to 3. The second file contains uncropped images for Fig 2A. The data that support the findings of this study are available from the corresponding author upon reasonable request.

Field-specific reporting

Please select the best fit for your research. If you are not sure, read the appropriate sections before making your selection.

☒ Life sciences ☐ Behavioural & social sciences ☐ Ecological, evolutionary & environmental sciences

For a reference copy of the document with all sections, see nature.com/authors/policies/ReportingSummary-flat.pdf

Life sciences study design

All studies must disclose on these points even when the disclosure is negative.

Sample size	No sample size calculation was performed. The background for chimerism and tolerance in our system is essentially zero, any meaningful chimerism is ~1%. Therefore, we estimated that 3 animals per group would be sufficient to see a meaningful difference. Instead we observed ~20% average chimerism using CD117 immunotoxin conditioning, indicating a very large effect.
Data exclusions	No data were excluded.
Replication	Each experiment was performed twice, each with large effects.
Randomization	Randomization is not relevant to this study. We are comparing various treatments to the same strain of mice.
Blinding	Each animal was given an ear tag number. Data were acquired and linked to the tag number and decoded after acquisition.

Reporting for specific materials, systems and methods

Materials & experimental systems

n/a	Involved in the study
☒	☐ Unique biological materials
☐	☒ Antibodies
☒	☐ Eukaryotic cell lines
☒	☐ Palaeontology
☐	☒ Animals and other organisms
☒	☐ Human research participants

Methods

n/a	Involved in the study
☒	☐ ChIP-seq
☐	☒ Flow cytometry
☒	☐ MRI-based neuroimaging

Antibodies

Antibodies used

For CD117 immunotoxin construction & HSC depletion:
Marker Color Clone # Vendor Cat# Lot#
c-kit/CD117 biotin 2B8 Biolegend 105804 170777-31

For transient immunosuppression:
Marker Color Clone # Vendor Cat# Lot#
CD4 YTS177 Bioxcell BE0003-3 669218M1
CD8 YTS169 Bioxcell BE0117 650018F1
CD40L MR-1 Bioxcell BE0017-1 627317J2

For FACS analysis:
Marker Color Clone # Vendor Cat# Lot#
H2Kd FITC SF1-1.1 Biolegend 116606 B175000
Ter119 AF700 Ter-119 Biolegend 116210 B247524
CD11b APC M1/70 Biolegend 101212 B261578
Gr-1 PE/Cy7 RB6-8C5 Biolegend 108416 B248638
CD19 APC/Cy7 6D5 Biolegend 115530 B228154
CD3 PB 17A2 Biolegend 100214 B227246
CD49b(PAN-NK) PE DX5 Biolegend 108908 B223922
Sca1 APC/Cy7 D7 Biolegend 108126 B227481
Flt3/CD135 APC A2F10 Biolegend 135312 B209576

IL7ra BV 605   A7R34 Biolegend 135041 B225950
c-kit/CD117 APC 2B8 Biolegend 105812 B217855
Lin PB Biolegend 133310 B225582
CD150 PE/Cy7   TC15-12F12.2 Biolegend 115914 B210492
CD34 biotin RAM34 BD 13-0341-85 E02498-1632

For HSC depletion:
Marker Color Clone # Vendor Cat# Lot#
c-kit/CD117 N/A ACK2 Biolegend 135101 N/A

Validation

For anti-CD117/ckit: reported in vivo transient removal of >98% of endogenous HSCs in immunodeficient mice; Science 318(5854): 1296–1299

The following validations are stated by the vendor:
For anti-CD4/YTS177: reported in vivo blockade of CD4+ T cell response; Tanspl Immunol 33(2): 125-129
For anti-CD8/YTS169: reported in vivo CD8+ T cell depletion; Nature 521 (7550):99-104
For anti-CD40L/MR-1: reported in vivo blockade of CD40/CD40L signaling; Nature Commun 6:7566

Validation statement of FACS Ab vendor:
Each lot of each antibody is quality control tested by immunofluorescent staining with flow cytometric analysis. Detailed information is available at the links below:
Marker Color Clone # Source Link
H2Kd FITC SF1-1.1 Ms http://www.biolegend.com/fitc-anti-mouse-h-2kd-antibody-1860.html
Ter119 AF700 Ter-119 Rat https://www.biolegend.com/en-us/search-results/alexa-fluor-700-anti-mouse-ter-119-erythroid-cells-antibody-3428
CD11b APC M1/70 Rat http://www.biolegend.com/apc-anti-mouse-human-cd11b-antibody-345.html
Gr-1 PE/Cy7 RB6-8C5 Rat http://www.biolegend.com/pe-cy7-anti-mouse-ly-6g-ly-6c-gr-1-antibody-1931.html
CD19 APC/Cy7 6D5 Rat http://www.biolegend.com/apc-cy7-anti-mouse-cd19-antibody-3903.html
CD3 PB 17A2 Rat http://www.biolegend.com/pacific-blue-anti-mouse-cd3-antibody-3317.html
CD49b(PAN-NK) PE DX5 Rat http://www.biolegend.com/pe-anti-mouse-cd49b-pan-nk-cells-antibody-234.html
Sca1 APC/Cy7 D7 Rat http://www.biolegend.com/apc-cy7-anti-mouse-ly-6a-e-sca-1-antibody-6752.html
Flt3/CD135 APC A2F10 Rat https://www.biolegend.com/en-us/products/apc-anti-mouse-cd135-antibody-6284
IL7ra BV 605   A7R34 Rat http://www.biolegend.com/brilliant-violet-605-anti-mouse-cd127-il-7ralpha-antibody-8539.html
c-kit/CD117 APC 2B8 Rat https://www.biolegend.com/en-us/search-results/brilliant-violet-711-anti-mouse-cd117-c-kit-antibody-12049
Lin PB Rat http://www.biolegend.com/pacific-blue-anti-mouse-lineage-antibody-cocktail-7765.html
CD150 PE/Cy7   TC15-12F12.2 Rat http://www.biolegend.com/pe-cy7-anti-mouse-cd150-slam-antibody-3056.html
CD34 biotin RAM34 Rat http://www.bdbiosciences.com/us/applications/research/stem-cell-research/cancer-research/mouse/pe-rat-anti-mouse-cd34-ram34/p/551387
c-kit/CD117 biotin 2B8 & Rat https://www.biolegend.com/en-us/products/biotin-anti-mouse-cd117-c-kit-antibody-73
c-kit/CD117 N/A ACK2 Rat https://www.biolegend.com/en-us/products/purified-anti-mouse-cd117-c-kit-antibody-6140

Animals and other organisms

Policy information about studies involving animals; ARRIVE guidelines recommended for reporting animal research

Laboratory animals

Mus musculus; males; C57Bl/6, Balb/c and CBA/Ca; 8-12 weeks of age at time of bone marrow transplantation

Wild animals

n/a

Field-collected samples

n/a

Flow Cytometry

Plots

Confirm that:

☒ The axis labels state the marker and fluorochrome used (e.g. CD4-FITC).

☒ The axis scales are clearly visible. Include numbers along axes only for bottom left plot of group (a 'group' is an analysis of identical markers).

☒ All plots are contour plots with outliers or pseudocolor plots.

☒ A numerical value for number of cells or percentage (with statistics) is provided.

Methodology

Sample preparation

Leukocytes were isolated from the blood, thymus, spleen, lymph node, BM, liver and lung from mice that had received BM transplantation. After lysing red blood cells, the cells were washed and incubated for 20 min with Fc blocking antibody (Clone 93, 1:50 BioLegend, San Diego, CA) followed by the relevant antibody combinations (1:50 dilution) and FACS staining buffer for 30 min at 4◦C.

Instrument	All data were collected using an LSRII flow cytometer (BD Biosciences)
Software	All data were analyzed with FlowJoTM 10 software (version 10.2; Treestar, Ashland, OR).
Cell population abundance	n/a: Leukocytes were not sorted.
Gating strategy	Details of gating strategy is shown in the figures of supplementary information.

☒ Tick this box to confirm that a figure exemplifying the gating strategy is provided in the Supplementary Information.
